# Surgical Outcome of Giant Goiters: A Retrospective Observational Study

**DOI:** 10.7759/cureus.115726

**Published:** 2026-09-03

**Authors:** Adarsh P Singh, Brijesh K Singh, Makwaana J Rahulbhai, Asuri Krishna, Sushant Soren, Seenu Vuthaluru

**Affiliations:** 1 Department of Surgical Disciplines, All India Institute of Medical Sciences, New Delhi, New Delhi, IND

**Keywords:** giant goiter, goiter, postoperative complications, thyroid, toatal thyroidectomy

## Abstract

Introduction: Surgical management of giant goiters is often challenging due to distorted anatomy and proximity to vital structures in the neck.

Objective: We aimed to evaluate the outcomes of patients operated on for giant goiters (size >10 cm) compared to non-giant ones (size ≤10 cm).

Methods: This retrospective study included 132 patients undergoing total thyroidectomy for various etiologies over 36 months (January 2022 to December 2024) in a single surgical unit of a tertiary care hospital. Clinical and surgical management details were noted from the electronic database and medical records. Data were analyzed by IBM SPSS Statistics for Windows, version 27 (IBM Corp., Armonk, NY, USA).

Results: Outcomes of 36 (27.2%) patients operated on for giant goiters were compared with 96 (72.8%) cases of non-giant goiters. Though the median duration of symptoms was significantly longer in patients with giant goiters than in those with non-giant goiters (66.0 (24.0-172.0) vs. 24.0 (8.0-69.0) months; p=0.005), the frequency of compressive symptoms was comparable between the two groups. Mean±SD size (cm) of giant goiters was 13.8±3.6, significantly higher than the size of non-giant goiters (7.2±1.6). Malignant pathology was significantly less frequent in patients with giant goiters than in those with non-giant goiters (44.4% vs. 67.8%; p = 0.039). Median duration of surgery, intraoperative blood loss, and duration of hospital stay were significantly higher for patients with giant disease. Although mean postoperative day-1 serum calcium levels were comparable between groups, the need for postoperative mechanical ventilation (16.7% vs. 1.0%; p = 0.002), tracheostomy (13.9% vs. 1.0%; p= 0.006), and postoperative recurrent laryngeal nerve palsy (13.9% vs. 4.2%; p = 0.048) was significantly higher in patients with giant disease. Other postoperative complications, including hypocalcemia rates, were similar.

Conclusion: Giant goiters were associated with greater intraoperative blood loss and higher frequencies of postoperative mechanical ventilation, tracheostomy, and recurrent laryngeal nerve palsy, while the incidence of hypocalcemia was comparable between the groups.

## Introduction

Thyroidectomy for goiters is one of the most common endocrine surgeries performed all over the world [1]. Due to proximity to the vital structures, thyroid surgeries are often associated with complications such as hemorrhage (0-4.2%), airway obstruction (0.1 - 0.3%), transient recurrent laryngeal nerve (RLN) injury (5-8%), permanent RLN palsy (0.3-3.0%) and transient (1.2-4.0%) and permanent hypocalcemia (3%) [2]. Though goiters are common, giant goiters are uncommon and pose a significant challenge during surgeries, as mentioned in several case reports and series [3-11]. Only a few large studies have compared the outcomes of giant and non-giant goiters [12,13].

Giant goiters have been defined variably in the literature based on the presence of compressive symptoms such as dyspnea, dysphagia, and hoarseness or based on weight and size of the thyroid gland. According to Du et al., a thyroid weighing >500 g or with a size of more than 10 cm, extending superiorly up to the mandibular angle, inferiorly up to the sternum, and laterally up to the posterior border of the sternocleidomastoid muscle is referred to as giant goiter [14].

Classification of goiters as giant or non-giant is important from the point of view of surgery because traditionally, the size of the gland is considered an important predictor of complications of thyroidectomy [15,16], whereas recent studies have refuted this notion [17]. Another important factor determining surgical complications of thyroidectomy is the surgeon’s volume and primary versus recurrent surgery [18]. Despite the endemicity of goiter in our country and improvement in postoperative care, there is a dearth of studies comparing outcomes of giant goiters in the contemporary era. The present retrospective study evaluated the surgical outcomes of giant versus non-giant goiters operated on for the first time in a high-volume center.

## Materials and methods

Methods

The present retrospective observational study was conducted in a single unit of the Department of Surgical Disciplines of a tertiary care teaching institute in Northern India over a period of 36 months (January 2022 - December 2024).

Study population

Patients undergoing total thyroidectomy for the first time comprised the study population. For the purpose of this study, giant goiter was operationally defined as a goiter measuring >10 cm in maximum dimension. Outcomes of patients with giant goiters were compared with those with non-giant goiters (size ≤10 cm).

Data collection

Clinical details, diagnostic workup, radiological findings, surgical management and outcomes, and duration of hospital stay of total thyroidectomies were recorded from the electronic database and patient records in a predesigned proforma. Details of the surgery such as operative time and intraoperative blood loss were also noted.

Peri-operative management

Standard guidelines for the surgical management of goiters were followed [19]. Patients were operated on by two high-volume surgeons (>50 thyroidectomies per year) of the unit. Both surgeons operated on patients in both the giant and non-giant goiter groups, with cases allocated according to routine clinical practice. All the patients underwent measurement of thyroid function tests, anti-thyroid-peroxidase antibodies, serum calcium and parathyroid hormone levels preoperatively and postoperatively on day 1, and vitamin D as part of routine preoperative workup. Examination of vocal cord movement by indirect laryngoscopy was performed prior to surgery in all the cases and after surgery in those developing hoarseness. During the intraoperative period, recurrent laryngeal nerve identification was done routinely in all the cases. Thyroid and adjacent anatomy was assessed preoperatively by ultrasound as well as CT scan in all the cases of giant goiters and suspected malignancy in cases with non-giant disease. Tissue diagnosis by fine needle aspiration cytology was obtained in all cases.

Outcomes

Given the retrospective observational design of the study, no primary outcome was prespecified. Only complications explicitly documented in the patient records were considered as postoperative outcomes. These included postoperative respiratory complications requiring mechanical ventilation or tracheostomy, hypocalcemia, recurrent laryngeal nerve palsy, and postoperative hemorrhage, where documented. In addition, duration of surgery and hospital stay were noted. Postoperative respiratory complications were defined as the need for mechanical ventilation beyond the expected immediate postoperative period or the requirement for tracheostomy. Hypocalcemia was defined as serum calcium <8 mg/dL and managed with intravenous and oral calcium supplementation and vitamin D. Postoperative RLN palsy was defined as vocal-cord paresis or paralysis detected on postoperative laryngoscopy, usually within the first few days/weeks.

Statistical analysis

Data were analyzed by IBM SPSS Statistics for Windows, version 27 (IBM Corp., Armonk, NY, USA). Quantitative data with a normal distribution were compared using the Student t-test, and those with a skewed distribution were analyzed using the Mann-Whitney U test. Proportions were compared using the Chi-square or Fisher's exact test as applicable. All the tests were two-tailed at a significance level of <0.05.

## Results

A total of 246 thyroidectomies were performed during the study period, of which 132 (53.8%) were primary total thyroidectomies and were included in the study. Cases of hemithyroidectomy and redo thyroidectomy were excluded to ensure a homogeneous study population (Figure 1).

**Figure 1:**
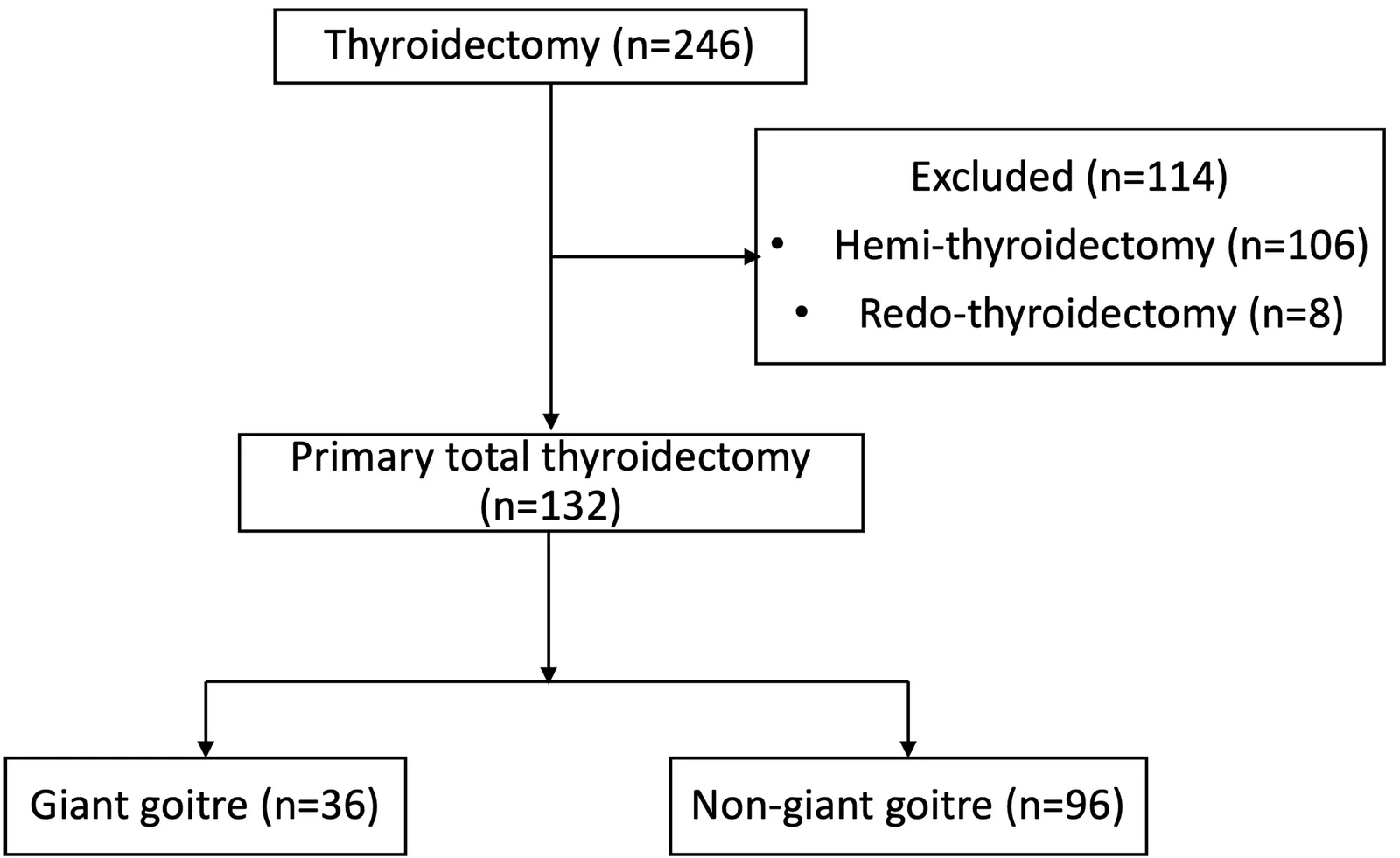
Study flow chart

Of the included patients, 36 (27.2%) had giant goiters, and the rest had non-giant goiters. The baseline characteristics of the patients are displayed in Table 1. 

**Table 1:** Baseline characteristics of the patients undergoing total thyroidectomy (a) Student's t-test; (b) Chi-square test; (c) Mann-Whitney U test; (d) Fisher's exact test IQR: interquartile range; SD: standard deviation

Parameters	Giant goiters (n=36)	Non-giant goiters (n=96)	p-value
Age (yr), mean ± SD	47.2 ± 13.4	42.8 ± 14.6	0.119^a^
Age < 50 yr, n (%)	19 (52.8)	70 (72.9)	0.028^b^
Males, n (%)	7 (19.4)	22 (22.9)	0.668^b^
Size of thyroid (cm), mean ± SD	13.8 ± 3.6	7.2 ± 1.6	<0.001^a^
Weight of thyroid (g), median (IQR)	245.0 (104.8 – 370)	58.0 (28.0 - 79.8)	<0.001^c^
Symptoms duration (m), median (IQR)	66.0 (24.0 – 172.0)	24.0 (8.0 – 69.0)	0.005^c^
Preoperative compressive symptoms, n (%)	9 (25.0)	18 (18.8)	0.428^b^
Retrosternal extension, n (%)	2 (5.6)	2 (2.1)	0.299^d^
Thyroid function status, n (%)	-	-	0.065^b^
Euthyroid	20 (55.6)	71 (74.7) (N=95)	-
Hypothyroid	12 (33.3)	17 (17.8)	-
Hyperthyroid	4 (11.1)	7 (7.5)	-

The clinical photograph of one of the patients with giant goiter managed during the study period is shown in Figure 2.

**Figure 2:**
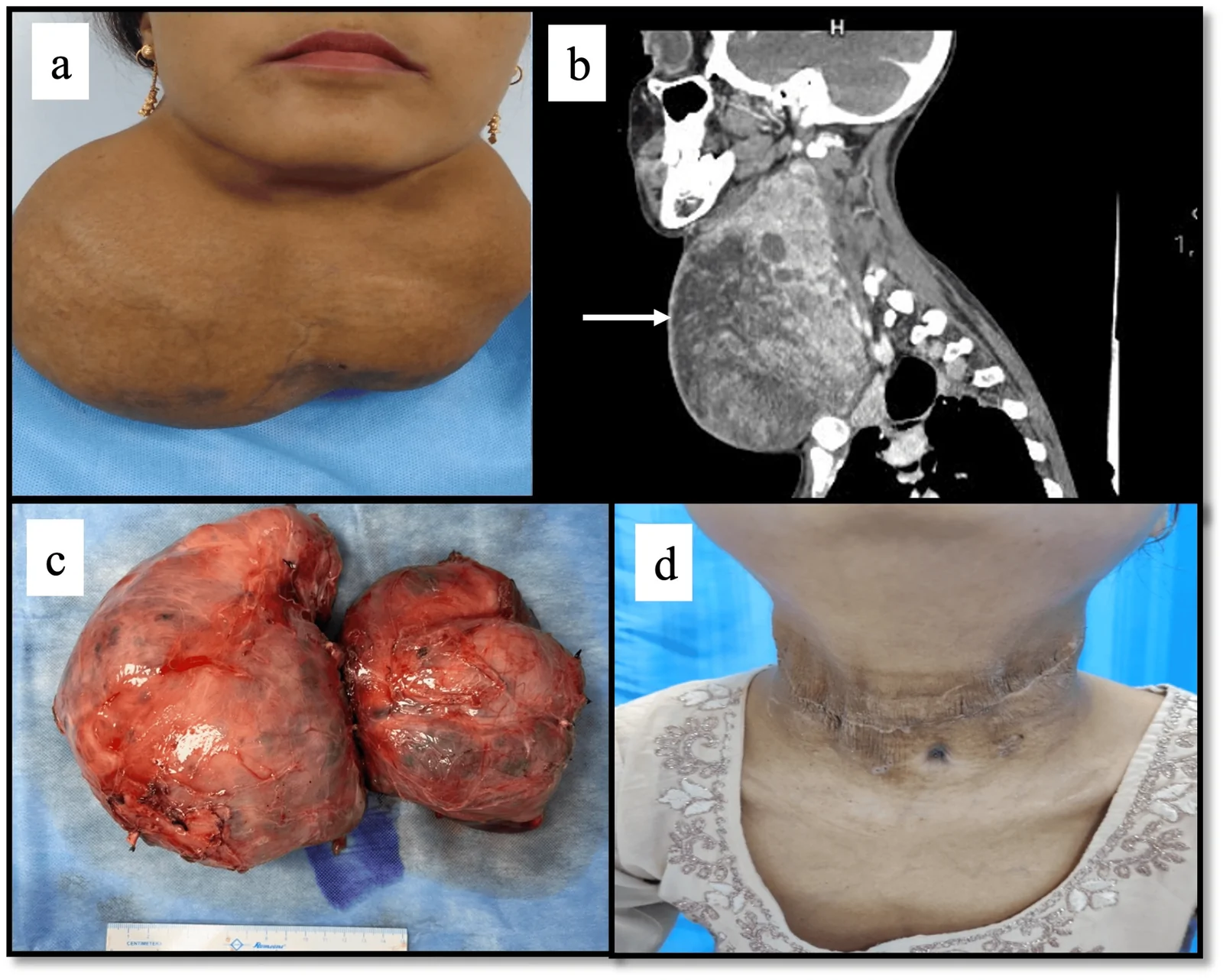
Preoperative and postoperative clinical photographs, computed tomography (CT) image of the neck, and gross specimen of the thyroid in a patient with a giant goiter (a) Preoperative image of a 27-year-old female patient with giant goiter; (b) Contrast-enhanced CT scan showing large thyroid swelling (white arrow) involving both the lobes and isthmus with encasement of trachea without any local invasion; (c) Specimen image with tumor size 25x15 cm, weighing 1.6 kg, (d) Postoperative image showing scar

The mean size of the goiter in the giant group was significantly higher than in the non-giant group (mean ± SD size (cm) of giant vs. non-giant goiter: 13.8 ± 3.6 vs. 7.2 ± 1.6, respectively; p<0.001). Median (IQR) weight (g) of the gland was also significantly higher in the giant group. Mean age of the two groups was comparable, whereas the duration of symptoms was significantly higher in the giant group. There was no significant difference between the groups regarding the occurrence of compressive symptoms. Benign disease comprised a significantly higher proportion of giant goiters.

Details of surgery and postoperative complications have been mentioned in Table 2.

**Table 2:** Comparison of surgical treatment and outcome (a) Student's t-test; (b) Chi-square test; (c) Mann-Whitney U test; (d) Fisher's exact test CCLND: central compartment lymph node dissection; IQR: interquartile range; MRND: modified radical neck dissection; POD: postoperative day; RLN: recurrent laryngeal nerve; SD: standard deviation; TT: total thyroidectomy

Parameters	Giant goiters (n=36)	Non-giant goiters (n=96)	p-value
Type of surgery, n (%)	-	-	0.427^b^
TT only	28 (77.8)	71 (74.0)	-
TT + CCLND	2 (5.6)	11 (11.5)	-
TT + unilateral MRND	3 (8.3)	3 (3.1)	-
TT + bilateral MRND	3 (8.3)	11 (11.5)	-
Duration of surgery (hr), median (IQR)	2.0 (1.5 – 2.5)	1.5 (1.5 – 2.0)	<0.001^c^
Blood loss (mL), median (IQR)	150.0 (100.0 – 200.0)	50.0 (50.0 – 100.0)	<0.001^c^
No of parathyroids identified, n (%)	-	-	0.426^b^
1-2	6 (16.7)	11 (11.5)	-
3-4	30 (83.3)	85 (88.5)	-
POD-1 serum calcium (mg/dL), mean ± SD	8.2 ± 0.8	8.3 ± 0.7	0.253^a^
POD-1 iPTH (pg/mL), median (IQR)	20.0 (12.8 – 33.0)	18.0 (8.8 – 26.6)	0.401^c^
Postoperative complications, n (%)	-	-	-
Hypocalcemia	4 (11.1)	24 (25.0)	0.082^b^
Need for mechanical ventilation	6 (16.7)	1 (1.0)	0.002^d^
Tracheostomy	5 (13.9)	1 (1.0)	0.006^d^
Thoracic duct injury	1 (2.8)	2 (2.1)	1.000^d^
Hematoma	1 (2.8)	2 (2.1)	1.000^d^
Sternotomy	0	2 (2.1)	1.000^d^
Postoperative recurrent laryngeal palsy, n (%)	5 (13.9)	4 (4.2)	0.048^d^
Duration of hospital stay (days), median (IQR)	3.0 (2.0 – 5.0)	2.0 (2.0 – 4.0)	0.009^c^
Final histopathology, n (%)	-	-	-
Benign disease	20 (55.6)	31 (32.2)	0.014^b^
Thyroid malignancy, n (%)	16 (44.4)	65 (67.8)	0.039^b^
Papillary thyroid carcinoma	12 (75.0) (N=16)	57 (87.7) (N=65)	0.200^b^
Follicular thyroid carcinoma	2 (12.5) (N=16)	8 (12.3) (N=65)	0.983^b^
Medullary thyroid carcinoma	2 (12.5) (N=16)	0 (N=65)	0.004^b^

Total thyroidectomy alone was performed in 28 (77.8%) and 71 (74%) of the patients with giant and non-giant goiters, respectively. The remaining eight (22.2%) cases of giant goiter and 25 (26.0%) of non-giant goiter underwent some form of lymph node dissection of the neck along with the thyroidectomy operation. Median duration of surgery and intraoperative blood loss were significantly higher in the giant group. Although the need for postoperative mechanical ventilation and tracheostomy was greater in the giant goiter group, other postoperative complications, including local-site hematoma and hypocalcemia, were similar between the groups. Postoperative RLN palsy occurred more frequently in patients with giant disease. Median duration of hospital stay was significantly higher for the patients operated on for giant goiter (median (IQR) duration of hospital stay (days), giant vs. non-giant: 3.0 (2.0 - 5.0) vs. 2.0 (2.0 - 4.0), p=0.009).

## Discussion

The present study compared clinical characteristics and postoperative outcomes of patients with giant and non-giant goiter. The duration of symptoms was significantly longer in patients with giant disease, with no significant difference in the distribution of compressive symptoms. Postoperative serum calcium levels and rate of hypocalcemia were comparable between the groups. Nearly three-fourths of the patients could be managed with total thyroidectomy alone in both groups; however, duration of surgery, intraoperative blood loss, need for mechanical ventilation and tracheostomy after surgery, duration of hospital stays, and rate of postoperative RLN palsy were significantly higher for giant cases.

In the present study, we have included patients operated on for the first time because surgical challenges are more in re-do settings [20]. More patients in the giant group manifested at a later age with a longer duration of symptoms, as observed in previous studies [12,13]. Goiters are more common in iodine-deficient endemic regions, where limited healthcare infrastructure, financial constraints, and poor health-seeking behavior may delay diagnosis and treatment, allowing the glands to progressively enlarge and reach massive sizes [10].

Compressive symptoms affected approximately 20% of the patients in both groups in our study, whereas in previous large studies, compressive features affected 50-80% of the patients with giant goiters. This could be attributed to the inclusion of re-do thyroidectomies in 12.4% and 32.4% of the cases in giant and non-giant groups, respectively, in the study by Chen et al. [13]. Only two cases in each group had retrosternal extension, possibly explaining the lower rate of compressive symptoms in our study [21]. Moreover, symptoms of compression depend not only on gland size but also on factors such as the direction of enlargement, retrosternal extension, anatomical relationships, rate of growth, and individual adaptation to gradual enlargement.

The rate of malignancy was significantly higher in the non-giant group in our study, whereas it was nearly one-third in both groups in the study by Agrawal et al. [12]. To the contrary, in another study [13], the malignancy rate was significantly higher in the giant group. Vrinceanu et al. had also found a malignancy rate of 71.43% in their 21 cases of giant goiters [22].

In our study, the majority of the patients could be managed with total thyroidectomy alone, with more intraoperative blood loss and longer duration of surgery in the giant group. Only one case of non-giant goiter needed sternotomy, as retrosternal extension was infrequent in both groups. Median duration of surgery was two hours and one-and-a-half hours in the giant and non-giant groups, respectively. Patoir et al. have also reported similar operative time for large goiters in their study [23]. Only one and two patients developed hematoma in the giant and non-giant groups, respectively, in our study. Previous studies also reported <5% risk of bleeding in either group [12,13].

Postoperative RLN palsy manifesting as hoarseness was found in 13.9% and 4.2% of the cases in our study, which was comparable to the previous studies [12,13]. The higher incidence of RLN palsy in patients with giant goiters may be related to distortion and displacement of the nerve by the enlarged gland, loss of normal anatomical planes, and increased difficulty in mobilizing the thyroid and identifying the nerve. These factors can increase the risk of inadvertent traction or thermal/mechanical injury during dissection. Rate of hypocalcemia occurred less often in the giant group in our study, though the difference was not statistically significant. Hypocalcemia occurred less frequently in our study than reported by Agrawal et al. [12]. The proportion of hypocalcemia was significantly higher in the massive goiters group (41.0%) compared to the large goiter group (25.0) in the study by Chen et al. [13]. The lower rate of hypocalcemia in our study could be explained by the identification of all four parathyroids in more than 80.0% of the cases in both groups.

Our study evaluated the outcomes of thyroidectomy for the relatively uncommon condition of giant goiters in the contemporary era. The findings highlight that giant goiters continue to be encountered in our setting despite ongoing national iodine deficiency control programs. Several limitations should be acknowledged. The retrospective, exploratory, single-center design and relatively small sample of patients with giant goiters (n = 36) limit generalizability and introduce the possibility of selection bias. No primary outcome was prespecified, and multiple outcomes were assessed without adjustment for multiple comparisons, potentially increasing the risk of type I error. The analysis was primarily unadjusted, and residual confounding cannot be excluded. Although mean age was comparable between groups, the distribution across age categories differed significantly, and age, along with other factors such as disease etiology, malignancy, and extent of surgery, may have influenced perioperative and postoperative outcomes. Information on intraoperative anesthetic challenges and long-term postoperative outcomes was not consistently available. RLN injury was assessed clinically, as objective confirmation with postoperative laryngeal electromyography or nerve conduction studies was not routinely available. Finally, although a one-dimensional size-based definition of giant goiter was selected for its simplicity, objectivity, and reproducibility using routinely available records, it may not fully reflect the three-dimensional anatomical complexity or cervical and mediastinal extent of the disease.

Overall, these findings provide contemporary real-world evidence regarding the surgical challenges and perioperative risks associated with giant goiters and support the need for meticulous preoperative evaluation, particularly of the airway and recurrent laryngeal nerves, in this patient population.

## Conclusions

To conclude, patients with giant goiters had significantly larger and heavier thyroid glands, a lower prevalence of malignancy, and a longer duration of symptoms before presentation compared with those with non-giant goiters. Giant goiters were also more frequently observed among older patients and were associated with higher rates of short-term postoperative complications, particularly the need for tracheostomy and postoperative mechanical ventilation. Postoperative RLN palsy was also more frequent in patients with giant goiters, even when surgery was performed by high-volume surgeons at a tertiary care center, possibly reflecting the technical challenges associated with distorted cervical anatomy. These findings highlight the importance of careful preoperative assessment and counseling, particularly regarding airway management and the potential for RLN injury, to facilitate appropriate surgical planning and perioperative preparedness.
